# Image processing for IMRT QA dosimetry

**DOI:** 10.1120/jacmp.v6i4.2071

**Published:** 2005-11-22

**Authors:** Mehran R. Zaini, Gary J. Forest, David D. Loshek

**Affiliations:** ^1^ Radiation Oncology Department Marshfield Clinic 1000 North Oak Avenue Marshfield Wisconsin 54449 U.S.A.

**Keywords:** radiation therapy, IMRT, QA, image processing

## Abstract

We have automated the determination of the placement location of the dosimetry ion chamber within intensity‐modulated radiotherapy (IMRT) fields, as part of streamlining the entire IMRT quality assurance process. This paper describes the mathematical image‐processing techniques to arrive at the appropriate measurement locations within the planar dose maps of the IMRT fields. A specific spot within the found region is identified based on its flatness, radiation magnitude, location, area, and the avoidance of the interleaf spaces. The techniques used include applying a Laplacian, dilation, erosion, region identification, and measurement point selection based on three parameters: the size of the erosion operator, the gradient, and the importance of the area of a region versus its magnitude. These three parameters are adjustable by the user. However, the first one requires tweaking in extremely rare occasions, the gradient requires rare adjustments, and the last parameter needs occasional fine‐tuning. This algorithm has been tested in over 50 cases. In about 5% of cases, the algorithm does not find a measurement point due to the extremely steep and narrow regions within the fluence maps. In such cases, manual selection of a point is allowed by our code, which is also difficult to ascertain, since the fluence map does not yield itself to an appropriate measurement point selection.

PACS numbers: 87.53.Xd, 87.66.Cd, 87.57.Nk

## I. INTRODUCTION

For every patient who undergoes intensity‐modulated radiotherapy (IMRT) treatment, our physics team performs a series of quality assurance (QA) tests. These tests are to ensure proper delivery of radiation dose to the appropriate geometrical sites within the patient.

Using our treatment‐planning system, Philips Pinnacle®, we produce multiple beams, each having varying radiation intensity patterns. Part of the QA process involves placing the generated fluence patterns on a flat‐water phantom in the planning software. Dose maps at two planes of 6 cm and 7 cm inside the phantom perpendicular to the beams are computed. One of the two depths (7 cm) is for absolute dosimetry verification at a single point, and the other one (6 cm) is for comparing the computed isodose patterns with those of the irradiated films. A spherical ion chamber (0.125 cm^3^) is used for the absolute measurements, and Kodak EDR‐2 films are used for relative dose maps. These measurements are performed during the same irradiation session for each beam. Absolute dosimetry with film is not as accurate and reproducible as it is with ion chamber. Our tolerance for absolute dosimetry accuracy is 5%. We are in the process of defining the limits of acceptance for comparing the isodose patterns, since it has so far been a qualitative comparison. For the isodose profile comparison, we are developing image subtraction software, as well as a system based on principal component analysis.

In addition to perpendicular dose planes at two different depths for each radiation beam, a computed transverse radiation map for the integral of all the beams is also compared with a measured pattern using a film. This transverse image is taken in either a cylindrical or thorax water phantom, which was designed in our department.

The entire IMRT QA procedure is time‐consuming for the physics team. We are devising a system that not only helps the physicists in performing the IMRT QA tasks more efficiently, but also allows the dosimetrist to be able to perform the IMRT QA measurement tasks with the medical physicist's supervision. This system incorporates the automation of some of the tasks that require trained eyes. One such automation task involves the determination of the optimum placement of a dosimeter (an ion chamber) within the intensity‐modulated field. A number of mathematical image processing techniques have been designed and tested in order to achieve this automation. This paper discusses the methods and results of the automated dosimetry localization system.

## II. METHODS

### A. Planar dose maps

The topology of planar dose maps (PDMs) often has regions with large gradients. Placing an ion chamber at such high‐gradient spots does not yield an accurate measurement. This is due to the finite size of the detector, as well as the imprecision in physical placement of the dosimeter at the intended position. If the detector is misplaced in the high‐gradient regions by 2 mm to 3 mm, the ion chamber measurements will be different than the computed values by more than our tolerance level of 5%. Furthermore, there are regions within the PDM that have a low magnitude (<15% of the maximum value), where the detector signal‐to‐noise ratio would be low, leading to measurement errors. Absolute measurements at a low magnitude area do not constitute a good test of the delivery system, since the high‐fluence regions deliver most of the dose to the patient, and their dose deposition accuracy should be verified.

It is difficult and time‐consuming to find such a position visually by inspecting the radiograph‐like images of the PDMs. On the other hand, automatically determining the location where an ion chamber measurement needs to be made is nontrivial. Mathematical image‐processing techniques allow the determination of such a position. In short, a specific region is identified based on its flatness, radiation magnitude, area, and the avoidance of the interleaf spaces of the multileaf collimators.

### B. Mathematical image processing

**Figure 1:**
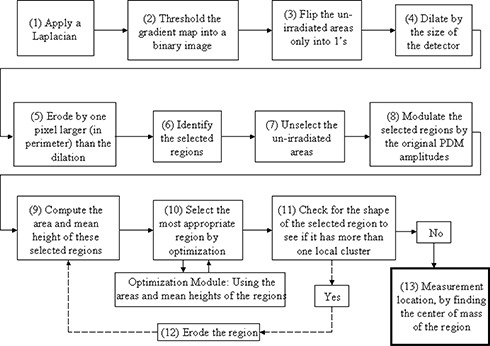
Mathematical image‐processing flowchart

Figure 1 depicts the various steps involved in determining the most suitable location for making dosimetric measurements in patients’ IMRT fields. MATLAB® by The Math Works, Inc.,© was used for the algorithm development in this work. The steps in Fig. 1 are explained below:

*Step 1*: A Laplacian is used on the planar dose images^(1)^:

(1) $$-\nabla^{2}f(x,y)=\frac{\partial^{2}}{\partial x^{2}}f(x,y)+\frac{\partial^{2}}{\partial y^{2}}f(x,y),$$

where *f* (*x, y*) is the two‐dimensional dose image as a function of spatial coordinates *x* and y.

For the sake of this work, we are using the word gradient instead of curvature, despite the fact that a Laplacian creates a curvature map. This oversight is justified by the method we used to extract the desired information from the processed images. The kernel associated with this Laplacian we have used to be convolved with the image is

(2) $$\left[\begin{array}{ccc} -1 & -1 & -1\\ -1 & 8 & -1\\ -1 & -1 & -1 \end{array}\right].$$

**Figure 2:**
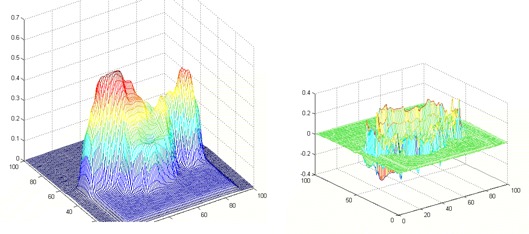
The original PDM and its Laplacian‐filtered image

Since the Laplacian is a second derivative operator, it will produce an abrupt zero crossing at an edge. The Laplacian is a linear, shift‐invariant operator, and its transfer function is zero at the origin of frequency space. Hence, a Laplacian‐filtered image will have zero mean gray level.^(1)^ In our case, we are interested in a narrow band around zero, as explained later, and not the zero crossings themselves. An example of a PDM and its Laplacian‐filtered image is shown in Fig. 2.

For a noise‐free image with sharp edges, a binary image formed by thresholding at the zero‐crossings of a Laplacian‐filtered image will produce closed‐contoured regions of interest (ROIs). In our case, since we seek gradient maps and no sharp edges normally exist, thresholding at a clinically relevant value is appropriate. Furthermore, for an image with noise, presmoothing with a Gaussian filter is the usual practice. However, applying a low‐pass filter prior to using the Laplacian is not desirable for our application, since the fluctuations in PDMs are desired variations and not unwanted noise. Hence, we simply apply a single Laplacian to the images.

*Step 2*: The threshold value that worked well was determined to be at ±0.02. This value is the relative gradient value in the Laplacian applied image. Even though this threshold value is adjustable, it rarely needs to be changed. The tuning range for this threshold value is ±0.01 to ±0.025, where ± is applied within the code. Using this thresholding limit, a binary mask is created. Upon thresholding, the image background gets selected as an acceptable area as well. However, picking the un‐irradiated background areas as a ROI is problematic, since it creates challenging issues at the subsequent processing steps. These issues include (1) the elimination of the regions close to the edge of the field that can occur in the subsequent image processing steps and (2) the selection of the un‐irradiated areas as a viable option.

*Step 3*: Removing the background as a ROI cannot be made by simply flipping the binary mask, since the binary mask is based on the gradient map, and the edges have a low gradient. To do this, the perimeter of these relatively flat regions is found. Then, upon labeling them (a MATLAB function), we can suppress the background to become an unselected ROI.

*Step 4*: Afterward, upon applying a dilation and an erosion operation, we first reduce the number of single streaks and small, unconnected regions and then eliminate their remaining ones. General dilation is defined as^(1)^

(3) $$D=B⊕S=\{x,y|S_{x,y}\cap B\neq \emptyset \},$$

where *D* is the resultant binary image, *B* is the original image, and *S* is the set of points (*x, y*) such that if *S* is translated so that its origin is located at (*x, y*), then its intersection with *B* is not empty. In this case, *S* is a 3×3 unity matrix, which translates to a 0.56 cm^2^ area, since the pixel is $0.25\times 0.25\;{\text{cm}}^{2}$. The algorithm is flexible with the pixel size, and its performance has been found not to be dependent on the dose grid sizes that have been tested ($0.1\times 0.1\;{\text{cm}}^{2}$ to $0.5\times 0.5\;{\text{cm}}^{2}$).

*Step 5*: Similarly, general erosion is defined as^(1)^

(4) $$E=B⊗S=\{x,y\;|S_{x,y}\subseteq B\},$$

where E, which is the resultant binary image, is formed by eroding the original image *B* by *S* such that if *S* is translated so that its origin is located at (*x, y*), then it is completely contained within B. For the erosion operation, *S* is a 4×4 unity matrix, which translates to a 1.0 cm^2^ area. The sizes of *S* for the dilation and erosion operations were chosen based on the size of the ion chamber used for the measurements. The sizes of *S* for the two operations are adjustable by the user, but this is almost never required.

*Step 6*: These dilation and erosion operations lead to the desired, relatively flat ROIs being delineated and all other unwanted regions being eliminated. The final ROIs get automatically identified by finding their perimeters, and they are labeled with natural numbers.

**Figure 3:**
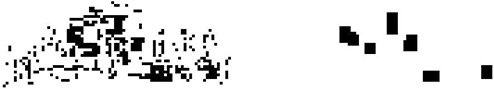
Laplacian image thresholded (left) and after applying dilation and erosion operations

*Step 7*: Since the background gets labeled again, it is suppressed for the second time. An example of a threshold‐applied image and its outcome after the dilation and erosion are shown in Fig. 3.

*Step 8*: In order to select the ROI that provides for both a reasonable magnitude and area, an optimization scheme is designed. This optimization process starts by modulating the ROIs by the original PDM.

*Step 9*: Mean height and area of the ROIs are computed to be used for the ROI selection. The ROI area is weighted less than its mean height in the optimization phase through a power parameter. This is because of the magnitude differences between the areas and mean heights, and because the heights of the ROIs are more important than their area for the ROI selection process.

*Step 10*: The power parameter was determined to be 0.2. The product of the rescaled mean height and the area to the power 0.2 is the objective function to be maximized:

(5) $$(\text{mean}\;\text{height})\times {\left(\text{area}\right)}^{0.2}.$$

This value is adjustable by the user as well, but in rare cases it needs to be changed to 0.25.

*Step 11*: The center of mass (CM) of the selected ROI is the desired position for ion chamber measurements. The CM computations include the details inside the ROI. In general, the geometric moment of order p+q for an image function *f* (*x, y*)is defined as^(2)^

(6) $$m_{pq}=\int_{-\infty }^{\infty }\int_{-\infty }^{\infty }x^{p}y^{q}f(x,y)\text{dxdy}.$$

The center of mass $\left(\bar{x},\bar{y}\right)$ is computed as

(7) $$\bar{x}=\frac{m_{10}}{m_{00}}\;\text{and}\;\bar{y}=\frac{m_{01}}{m_{00}},$$

where $m_{00}=N$, the total number of pixels inside the region.

However, it is possible that this CM falls into a narrow region of a ROI connecting two or more local clusters. In such cases, it is necessary to breakup the ROI into smaller regions. To do this, the smallest rectangle that encompasses the ROI is found. The ratio of the points inside the ROI and that of the bounding rectangle is computed. If this ratio is larger than 70%, it is reasonable to assume one main cluster, in which case the CM is the proper choice. However, if this ratio is less than 70%, a breakup of the ROI is warranted.

*Step 12*: Further eroding and labeling the subregions accomplish this breakup. The produced subregions need to be reevaluated against the previously found ROIs to determine the optimum ROI to select.

*Step 13*: Finally, the CM of the reselected ROI is chosen as the desired point of measurement.

## III. RESULTS AND DISCUSSION

**Figure 4:**
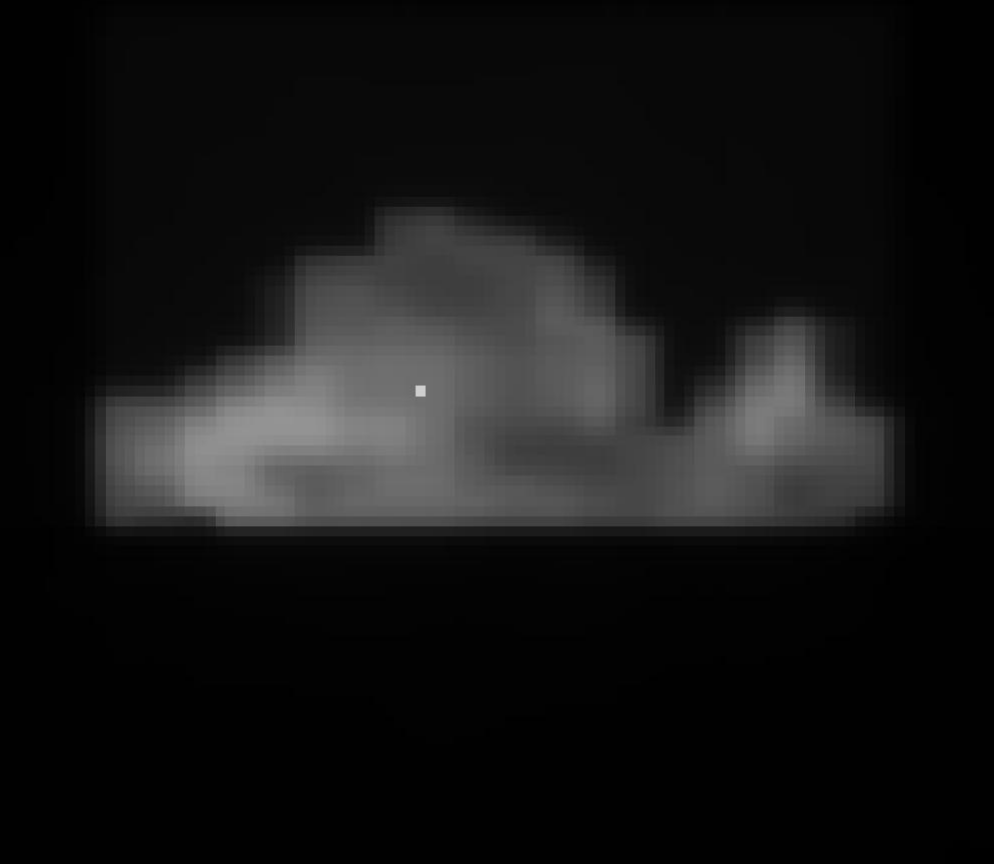
A PDM with the selected point of measurement

An example of a PDM image that depicts the automatically selected measurement point is displayed in Fig. 4. The selected point is accentuated in Fig. 4 for demonstration purposes only. It is apparent that determining the location of an appropriate measurement point visually is cumbersome. The coordinates of the identified point are passed to the user for the placement of the ion chamber in the phantom. The avoidance of the multileaf collimator interleaf spots is achieved using proper indexing of the results with respect to the actual leaf dimensions.

We have allowed for the possibility of failure of this code in extreme cases, in which cases the user can manually select a position for the ion chamber placement. In testing this algorithm in more than 50 cases, in only 5% of the beams one needs to resort to our manual point selection. The results of dose measurement agreements with Pinnacle predictions for a random sample of our IMRT patients are presented in Table 1. Even though it is possible to iteratively adjust the three parameters discussed earlier to achieve the point selection through this algorithm, the manual method is available if one does not wish to adjust any parameters. An automated parameter optimization algorithm is the focus of our future work.

**Table 1:** Dosimetry results based on automated and manual point selections

Patient	Number of IMRT Fields Automatic (A) Manual (M)	Measured versus Predicted by Pinnacle
1	A: 7	0.982. 0.990. 0.981, 1.001, 1.001. 0.977. 0.977
	M: 2	0.995, 0.999
2	A: 5	0.980, 0.956, 0.968, 0.984, 0.983
	M: 1	0.997
3	A: 9	0.967. 0.951. 0.966. 0.967. 0.937. 0.957. 0.970. 0.973. 0.939
	M: 0	—
4	A: 5	0.980, 0.984, 0.962, 0.979, 1.003
	M: 0	—
5	A: 5	1.048, 1.002. 0.977. 1.033, 1.073
	M: 0	—
6	A: 7	1.002. 0.990, 1.012. 1.009, 0.992. 0.974. 0.972
	M: 0	—
7	A: 10	0.955. 0.966. 0.982, 0.961, 0.976. 0.997. 0.982. 1.001. 0.973. 0.993
	M: 0	—

It seems reasonable to assume that pixels larger than $0.25\times 0.25\;{\text{cm}}^{2}$ would reduce the failure rate of this algorithm, but then high‐frequency intensity variations would be missed. Smaller dose computation grids, on the other hand, not only increase the PDM computation time; they would potentially lead to a higher failure rate of this code to find a suitable dosimetry localization point. Furthermore, a 0.125 cm^3^ ion chamber is a reasonable size of measurement device. This is because larger ion chambers will reduce the efficacy of IMRT QA, and the smaller ones will lead to enhanced measurement noise and more susceptibility to positional accuracy.

The manual point selection involves visually inspecting the promising areas, displaying the magnitude of the point under the mouse and the mean gradient for its eight‐neighbor pixels, and picking a point based on its magnitude and mean gradient. This selection is made by deciding on a point based on the compromise between a high magnitude and a low immediate neighbor gradient. Such a determination is made by observing a few promising regions. It is not based on an exhaustive search, since a complete search takes more than a few minutes per beam. The automatic code of this paper was developed precisely because a manual and visual point selection is time‐consuming, it is not as accurate, and it might not lead to the best measurement point.

We attempted to determine a measurement point using the tools available in Pinnacle. This process alone took more than 30 min, whereas our automated code achieves the same outcome in 2 min for all the IMRT beams of a patient. The process of using the Pinnacle tools involved taking the IMRT plan to a water phantom, repositioning the computation points of interest, reorienting the beams, recomputing the dose for all the beams, and creating the isodose lines in the plane of interest. Using the Pinnacle tools takes considerable training of the operator to get consistent results. One of the goals of this algorithm is to make the process less subjective. On the other hand, the planar dose computation window of Pinnacle does not allow one to create isodose lines on it. Furthermore, the window/level tool does not help one to inspect the PDM, in the manner it is displayed on the planar dose computation window. Henceforth, using Pinnacle for the purposes of this work is not appropriate.

## IV. CONCLUSION

The automated dosimetry localization technique discussed here simplifies and expedites the IMRT QA process for the medical physicist. It also allows the dosimetrist to perform the IMRT QA procedure almost independently. The clinical implementation of this system simply involves reading the PDM produced by Pinnacle into the code, and letting the automated code show you the measurement point. If the selected point is in a low magnitude region (as seen visually by its gray‐level magnitude compared to the rest of the image), one can adjust one or two of the parameters to arrive at a more reasonable point. In 95% of the cases, such parameter adjustment is not necessary. If one does not want to adjust the parameters, one has the option of selecting the point completely manually by just observing the magnitude and the local gradient under the motion of the mouse. Both methods establish suitable dosimetry verification points where measurements agree with computer predictions within our tolerance limit of 5% for correct IMRT plans.

We are working on automating more pieces of the IMRT QA process, such as quantitative film analysis, the creation of the lookup table for film dosimetry, and the incorporation of these pieces into our record‐and‐verify system. In addition, we are in the process of creating a more robust optimization module, where the parameter space is also searched automatically.

## ACKNOWLEDGMENTS

The authors thank Marshfield Clinic Research Foundation for its support through the services of Alice Stargardt in the preparation of this manuscript.

## Supporting information

Supplementary Material Files
